# Relationships between rDNA, Nop1 and Sir complex in biotechnologically relevant distillery yeasts

**DOI:** 10.1007/s00203-016-1258-9

**Published:** 2016-06-21

**Authors:** Jagoda Adamczyk, Anna Deregowska, Leszek Potocki, Ewelina Kuna, Jakub Kaplan, Sylwia Pabian, Aleksandra Kwiatkowska, Anna Lewinska, Maciej Wnuk

**Affiliations:** 1 Department of Genetics, University of Rzeszow, Rejtana 16C, 35-959 Rzeszow, Poland; 2 Postgraduate School of Molecular Medicine, Medical University of Warsaw, Warsaw, Poland; 3 Department of Biochemistry and Cell Biology, University of Rzeszow, Zelwerowicza 4, 35-601 Rzeszow, Poland

**Keywords:** Distillery yeasts, rDNA, Nucleolus, Nop1, Sirtuins

## Abstract

Distillery yeasts are poorly characterized physiological group among the *Saccharomyces sensu stricto* complex. As industrial yeasts are under constant environmental stress during fermentation processes and the nucleolus is a stress sensor, in the present study, nucleolus-related parameters were evaluated in 22 commercially available distillery yeast strains. Distillery yeasts were found to be a heterogeneous group with a variable content and length of rDNA and degree of nucleolus fragmentation. The levels of rDNA were negatively correlated with Nop1 (*r* = −0.59, *p* = 0.0038). Moreover, the protein levels of Sir transcriptional silencing complex and longevity regulators, namely Sir1, Sir2, Sir3 and Fob1, were studied and negative correlations between Sir2 and Nop1 (*r* = −0.45, *p* = 0.0332), and between Sir2 and Fob1 (*r* = −0.49, *p* = 0.0211) were revealed. In general, *S. paradoxus* group of distillery yeasts with higher rDNA pools and Sir2 level than *S. bayanus* group was found to be more tolerant to fermentation-associated stress stimuli, namely mild cold/heat stresses and KCl treatment. We postulate that rDNA state may be considered as a novel factor that may modulate a biotechnological process.

## Introduction

The major role of the nucleolus is ribosome biogenesis, but also other multiple functions have been assigned to the nucleolus (Boisvert et al. 2007; Pederson 1998). More recently, the nucleolus has been considered as a stress sensor (Grummt 2013; Lewinska et al. 2010; Mayer and Grummt 2005; Olson 2004). Mammalian nucleolus can sense and transmit oxidative and ribotoxic stress stimuli by downregulation of the rRNA synthesis by c-Jun N-terminal kinase 2 (JNK2)-mediated inactivation of an essential transcription factor TIF-IA (RRN3) modulating the activity of RNA polymerase I (Pol I) and saving the energy required to maintain cellular homeostasis during stress (Mayer et al. 2005). Numerous stress factors, e.g., heat shock, UV irradiation, hypoxia, DNA damaging agents and reactive oxygen species (ROS), may induce nucleolar stress that results in the elevation of the p53 level and cell cycle arrest and/or apoptosis by stress-induced relocation of nucleolar proteins from nucleolus to nucleoplasm/cytoplasm and changes in MDM2 (E3 ubiquitin ligase) activity (Mayer et al. 2004; Olson 2004; Rubbi and Milner 2003). Oxidant-induced nucleolar stress and the involvement of transcription factor Rrn3 have also been reported in the yeast *Saccharomyces cerevisiae* providing evidence for a common nucleolus-centered stress response in eukaryotic cells (Lewinska et al. 2010). Stress signals may also stimulate nucleolus fragmentation that is accompanied by the upregulation of nucleolar proteins, namely Nop1 and/or Nop2 (de Beus et al. 1994; Deregowska et al. 2015a; Lewinska et al. 2014a). Nop1 (fibrillarin) is required for pre-rRNA processing in yeast (Tollervey et al. 1991). More recently, Nop1 was reported to be a histone glutamine methyltransferase that modifies H2A at Q105 in nucleolus (Tessarz et al. 2014). rDNA may also promote genetic stability and regulate cellular stress response in industrially relevant yeast strains involved in winemaking and beer production (Deregowska et al. 2015a) that suggests that nucleolus state is an important parameter during microbe-based industrial fermentations at harsh environmental conditions.

There are two proteins that are implicated in the maintenance of rDNA copy number and rDNA stability as well as the regulation of yeast longevity, namely Fob1 and Sir2 (Defossez et al. 1999; Kaeberlein et al. 1999; Kobayashi 2006). rDNA copies may be recovered as a result of Fob1-mediated inhibition of replication fork progression at the replication fork barrier (RFB) promoting double-strand breaks and recombination-based amplification (Kobayashi 2006). In contrast, Sir2 silences a noncoding bidirectional promoter E-pro in the rDNA locus that inhibits the dissociation of the cohesion complex from rDNA and prevents the changes in rDNA copy number (Kobayashi 2006, 2008). Sir proteins, especially Sir2, a highly conserved NAD-dependent histone deacetylase, are well-recognized regulators of longevity in yeasts, worms and flies and also modulators of metabolic health in mammals (Covington and Bajpeyi 2016; Giblin et al. 2014; Kaeberlein et al. 1999). Sir2/3/4 complex was suggested to act indirectly to promote replicative lifespan by repressing transcription at *HML* and *HMR* (Kaeberlein et al. 1999). Sir2p may also act directly to suppress extrachromosomal rDNA circles (ERC) formation by inhibiting homologous recombination at a blocked replication fork in the rDNA, and decreased replicative lifespan of cells lacking active *SIR2* gene cannot be altered by deleting the *HM* loci (Kaeberlein et al. 1999).

Distillery yeasts used in food industry to produce distilled spirits such as vodka and whiskey are a group of industrial yeast strains that is poorly described (Deregowska et al. 2015b; Naumova et al. 2013). To date, a selected group of distillery yeast strains has been characterized genetically (*n* = 36) (Naumova et al. 2013) and genomically (*n* = 22) (Deregowska et al. 2015b). Distillery yeasts were assigned to the *S.cerevisiae* species and found to be aneuploid and rich in polymeric genes *SUC* and *MAL* important for sucrose and maltose fermentation, respectively (Naumova et al. 2013). In contrast, we have shown that a commercially available group of distillery yeast strains belonged to four species of the *Saccharomyces sensu stricto* complex (*S. bayanus*, *S. paradoxus*, *S. kudriavzevii* and *S. cerevisiae*) (Deregowska et al. 2015b). The diploid nature of distillery yeasts has also been revealed (Deregowska et al. 2015b). Array-based comparative genomic hybridization (array CGH) analysis showed that the variabilities in the gene copy number and *loci*-specific gains and losses involved mainly the subtelomeric regions and naturally occurring diversity in the *YRF1* (Y′ element ATP-dependent helicase) gene copy number may promote genetic stability in the *S. bayanus* group of distillery yeast strains (Deregowska et al. 2015b). However, more studies on the characteristic features of distillery yeasts, especially that of fundamental importance during industrial fermentations at stressful conditions, are needed.

In the present study, we have analyzed nucleolus parameters in 22 commercially available distillery yeast strains and revealed the relationships between rDNA pools, the levels of nucleolar protein Nop1, Sir transcriptional silencing complex and longevity regulators, namely Sir1p, Sir2p, Sir3p and Fob1p and the tolerance to fermentation-associated stress stimuli. We postulate that rDNA state may be considered a novel parameter affecting yeast fermentation performance.

## Materials and methods

### Chemicals

All reagents were obtained from Sigma (Poznan, Poland) unless otherwise specified.

### Yeast strains and growth conditions

All distillery yeast strains used in this work are listed in Table 1. Yeast from one single colony was grown either on liquid YPD medium (1 % w/v Difco Yeast Extract, 2 % w/v Difco Yeast Bacto-Peptone, 2 % w/v dextrose) or on solid YPD medium containing 2 % w/v Difco Bacto-agar, at 28 °C.

**Table 1:** Distillery yeast strains used in this study

No.	Trade name	Species name according to (Deregowska et al. 2015b)	Supplier
1	Samogon turbo	*S. bayanus*	CBF Drinkit
2	Superyeast T48 Dual Use	*S. bayanus*	CBF Drinkit
3	Spiritferm Extreme 8 kg Turbo	*S. bayanus*	Spiritferm
4	Spiritferm T3	*S. bayanus*	Spiritferm
5	Spiritferm turbo fruit	*S. bayanus*	Spiritferm
6	Spiritferm Moskva style	*S. bayanus*	Spiritferm
7	Coobra 24 Snabbsats	*S. paradoxus*	CBF Drinkit
8	Coobra 6 Magnum Snabbsats	*S. paradoxus*	CBF Drinkit
9	Coobra 8 Snabbsats	*S. cerevisiae*	CBF Drinkit
10	Coobra 48 Turbo Yeast	*S. paradoxus*	CBF Drinkit
11	Coobra RUM YEAST	*S. paradoxus*	CBF Drinkit
12	Double Snake Turbo Yeast C3 Extra	*S. paradoxus*	Hambleton Bard Ltd.
13	Alcotec Pure Turbo Super Yeast 48	*S. paradoxus*	Hambleton Bard Ltd.
14	Drożdże gorzelnicze Turbo 72 h	*S. paradoxus*	BROWIN
15	Black Bull Turbo Yeast	*S. paradoxus*	Avedore Trading
16	Gozdawa 1410 Turbo	*S. bayanus*	Gozdawa
17	Superyeast T Vodka Star	*S. paradoxus*	CBF Drinkit
18	Alcotec Vodka Star Turbo Yeast	*S. paradoxus*	Hambleton Bard Ltd.
19	Alcotec Single Strain Whisky with Amyloglucosidase	*S. kudriavzevii*	Hambleton Bard Ltd.
20	Fermiol drożdże gorzelnicze	*S. paradoxus*	BIOWIN/FERMIOL
21	BIOWIN Turbo Super Yeast 48 h	*S. paradoxus*	BIOWIN
22	Alcotec Pure Turbo Super Yeast 24 h	*S. paradoxus*	Hambleton Bard Ltd.

The strains have previously been assigned to the *Saccharomyces sensu stricto* complex (Deregowska et al. 2015b)

### rDNA analyses

rDNA was detected using Southern blotting using rDNA-specific probe (Lewinska et al. 2014a), fluorescence in situ hybridization (FISH) using whole chromosome XII painting probe (Wnuk et al. 2015) and polymerase chain reaction (PCR). DNA was extracted according to (Amberg et al. 2005). For Southern blotting-based analysis of rDNA length after DNA digestion with *BamH*I, rDNA-specific signals were detected using digoxigenin labeling, antidigoxigenin antibody and alkaline phosphatase based chemiluminescence using a G:BOX imaging system (Syngene, Cambridge, UK) (Lewinska et al. 2014a). Briefly, to create an rDNA-specific probe, the pNOY373 plasmid, a derivative of the high copy number plasmid YEp351 carrying rDNA with a promoter starting from −206 with a *XhoI*–*NotI* flanked enhancer, *LEU2*, 2 µ, *amp*, was used (Wai et al. 2000). pNOY373 DNA containing the 18S rRNA coding region (1 µg) was labeled with digoxigenin-11-deoxyuridine 5′-triphosphate (dUTP) using the DIG Nick Translation Mix (Roche) according to manufacturer’s instruction. rDNA length was quantified using GelQuantNET software (http://biochemlabsolutions.com/GelQuantNET.html) using the background correction option. The length of rDNA was calculated per amount of genomic DNA. For FISH, biotin-labeled chromosome XII-specific DNA was detected using Star*FISH© Biotin Painting Kit—FITC Label (Cambio, UK). Chromosome XII-specific signals were counted and presented as a percentage of 100 total cell scores. Moreover, to analyze the nucleolar rDNA content (chromosome XII-specific signals), ImageJ software (http://rsbweb.nih.gov/ij/) was used as described elsewhere (Lewinska et al. 2014b). Briefly, we evaluated the integrated fluorescence density (green channel), which is the sum of all pixel values within the marked area of each cell analyzed and equivalent to the product of the area and mean gray value. The integrated fluorescence density is presented in relative fluorescence units (RFUs). For PCR-based quantitative analysis of rDNA pools, REDTaq^®^ ReadyMix™ PCR Reaction Mix was used according to manufacturer’s instructions (R2523, Sigma). For amplification of the fragment of yeast 18S rRNA gene (*RDN18*-*2* gene) (1000 bp), DNA (125 ng), primers Fwd 5′-AGTTGCCCCCTTCTCTAAGC-3′ and Rev 5′-CCTTGAGTCCTTGTGGCTCT-3′ (Wnuk et al. 2009), and a total of 34 cycles of 30 s at 94 °C, 30 s at 60 °C, 30 s at 72 °C with an initial denaturation of 4 min at 94 °C and a terminal extension of 5 min at 72 °C were used. PCR products were visualized on a 1 % agarose gel and stained using ethidium bromide staining. DNA molecular size marker GelPilot Mid Range Ladder (100–2000 bp, Qiagen) was used. rDNA was quantified using GelQuantNET software (http://biochemlabsolutions.com/GelQuantNET.html). rDNA content is presented in arbitrary units (a.u.).

### Nucleolus morphology

To visualize the nucleolus, silver staining of nucleolar organizer regions (AgNOR) was performed. Silver staining of nucleolar argyrophilic proteins was conducted according to (Lewinska et al. 2014a). A total of 100 cells were analyzed, and their nucleolus morphological type was determined (unaffected or fragmented nucleolus) (%).

### Western blotting

For Western blotting analysis, whole cell extracts were prepared according to (Lewinska et al. 2014a). The following primary antibodies were used: anti-Nop1p (1:400), anti-Fob1p (1:200), anti-Sir1p (1:200), anti-Sir2p (1:200), anti-Sir3p (1:200) and anti-Act1p (1:1000) (Santa Cruz, Abcam). The respective proteins were detected after incubation with one of the horseradish peroxidase-conjugated secondary antibodies (1:80000, 1:100000 or 1:125000) (Sigma). The chemiluminescence signals were detected with a Clarity™ Western ECL Blotting Substrate (BioRad) and a G:BOX imaging system (Syngene, Cambridge, UK). Quantitative analysis of protein levels was conducted using GelQuantNET software (http://biochemlabsolutions.com/GelQuantNET.html). Protein content is presented in arbitrary units (a.u.).

### Utilization of non fermentable carbon sources and sensitivity to fermentation-associated stress stimuli

The spot assay (the semiquantitative measurement of growth/survival) (Lewinska et al. 2011) was used. To analyze the utilization of non fermentable carbon sources, a dilution of 1 × 10^5^ cells/ml of a yeast exponential phase culture in a volume of 2 μl was used, inoculated on solid YPG medium (1 % w/v Difco Yeast Extract, 2 % w/v Difco Yeast Bacto-Peptone, 2 % v/v glycerol) and YPE medium (1 % w/v Difco Yeast Extract, 2 % w/v Difco Yeast Bacto-Peptone, 2 % v/v ethanol) containing 2 % w/v agar, at 28 °C, and inspected after 48 h. For stress tolerance analysis, yeast cells were grown on standard solid YPD medium in the presence of NaCl, KCl and sorbitol (0.5, 1 and 1.5 M), high glucose concentrations (5, 10 and 20 %), ethanol (5 and 10 %) or at different temperature conditions (4, 20, 28, 37 and 55 °C). Hydrogen peroxide toxicity was analyzed after 40 min incubation of cells (1 × 10^7^ cells/ml) in the presence of 2, 5 and 10 mM H_2_O_2_ and transfer to solid YPD medium. Typically, the cell growth was inspected after 48 h.

### Statistical analysis

The results represent the mean ± SD from at least three independent experiments. The correlation analysis was performed using linear correlation (Pearson *r*) test using GraphPad Prism 5 (GraphPad Software, Inc., La Jolla, CA, USA). The differences between *S. bayanus* and *S. paradoxus* groups of distillery yeasts were assessed by Student’s *t* test using GraphPad Prism 5 (GraphPad Software, Inc., La Jolla, CA, USA).

## Results and discussion

**Fig. 1:**
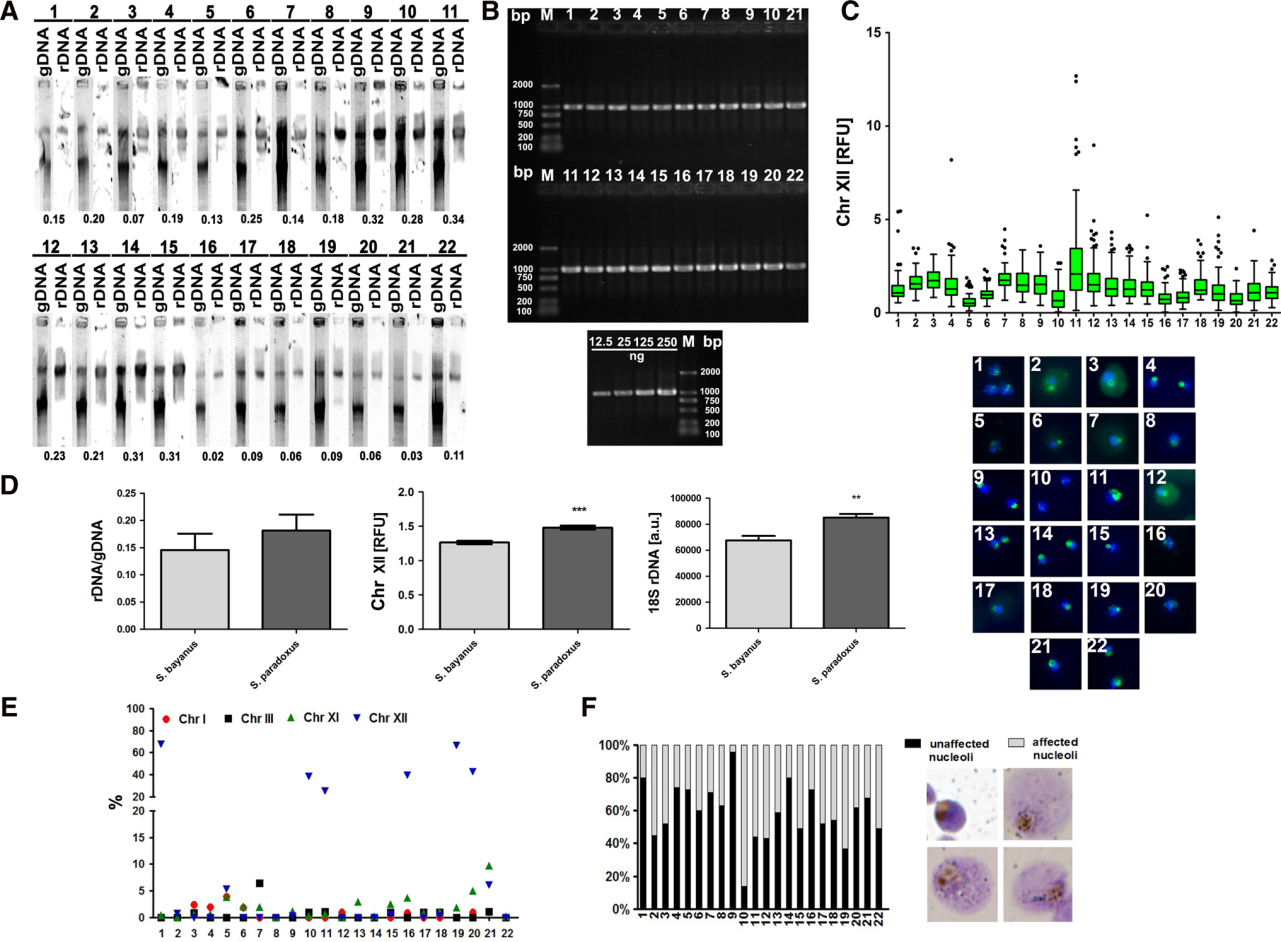
Analysis of selected nucleolus parameters in 22 distillery yeasts. **a** Southern blot analysis of the rDNA length. gDNA, genomic DNA after digestion with *BamH*I; *lanes* 1–22, 22 distillery strains. The length of rDNA calculated per amount of DNA is also shown (*bottom*). **b** Polymerase chain reaction (PCR)-based analysis of rDNA levels. *Lane* M, DNA molecular size marker; *lanes* 1–22, 22 distillery strains. Template amount-dependent PCR results are also shown (*bottom*). DNA from strain 10 (12.5, 25, 125 and 250 ng) was subjected to PCR-based analysis of rDNA content (*lanes* 12.5, 25, 125 and 250). **c** Fluorescence in situ hybridization (FISH)-based analysis of rDNA content. rDNA was visualized using whole chromosome painting probe (WCPP) specific to chromosome XII that contains rDNA locus in yeast (*green, bottom*). Fluorescence signals of chromosome XII were quantified using ImageJ software (*top*). The integrated fluorescence density is presented in relative fluorescence units (RFUs). The box-and-Tukey *whisker plots* are shown, *n* = 100. Typical micrographs are also presented (*bottom*). The cells were labeled with FITC to detect chromosome XII-specific signals (*green*). DNA was visualized using DAPI staining (*blue*). **d** Comparative analysis of rDNA pools between two distillery yeast groups, namely *S. bayanus* group (strains 1, 2, 3, 4, 5, 6 and 16) and *S. paradoxus* group (strains 7, 8, 10, 11, 12, 13, 14, 15, 17, 18, 20, 21 and 22) using Southern blotting (*left*), FISH (*middle*) and PCR (*right*). The *bars* indicate SD or SEM, *n* = 3, **p < 0.01, ***p < 0.001 compared to *S. bayanus* group (Student’s *t* test). **e** Analysis of chromosome I, III, XI and XII signals using fluorescence in situ hybridization (FISH) and whole chromosome painting probes (WCPPs). Chromosome-specific signals were scored in 100 nuclei and presented as a percentage; namely, more than two chromosome-specific signals are shown, *n* = 100. **f** Silver staining of nucleolar organizer region-based analysis of nucleolus fragmentation. Fragmented nucleoli were scored (%). The typical micrographs are also shown (*right*) (color figure online)

We have recently shown that rDNA acts as a genome buffer promoting chromosome homeostasis and regulates ethanol stress response in industrial yeast strains involved in winemaking and beer production (Deregowska et al. 2015a). Thus, we propose that rDNA state may be considered as a novel factor affecting overall fermentation performance during yeast-based biotechnological process (Deregowska et al. 2015a). In the present study, we have further investigated nucleolus parameters in 22 commercially available distillery yeast strains (Table 1). Distillery yeasts used in food industry to produce distilled spirits such as vodka and whiskey are not adequately characterized (Deregowska et al. 2015b; Naumova et al. 2013). Selected group of 22 distillery strains has already been classified by us into four *Saccharomyces* species categories, namely *S. bayanus* (*n* = 7), *S. cerevisiae* (*n* = 1), *S. kudriavzevii* (*n* = 1) and *S. paradoxus* (*n* = 13) belonging to the *Saccharomyces sensu stricto* complex using PFGE separation (molecular karyotyping) (Deregowska et al. 2015b). First, the length and levels of rDNA were investigated using three different methods, namely Southern blotting, FISH and PCR (Fig.1a–c).

**Fig. 2:**
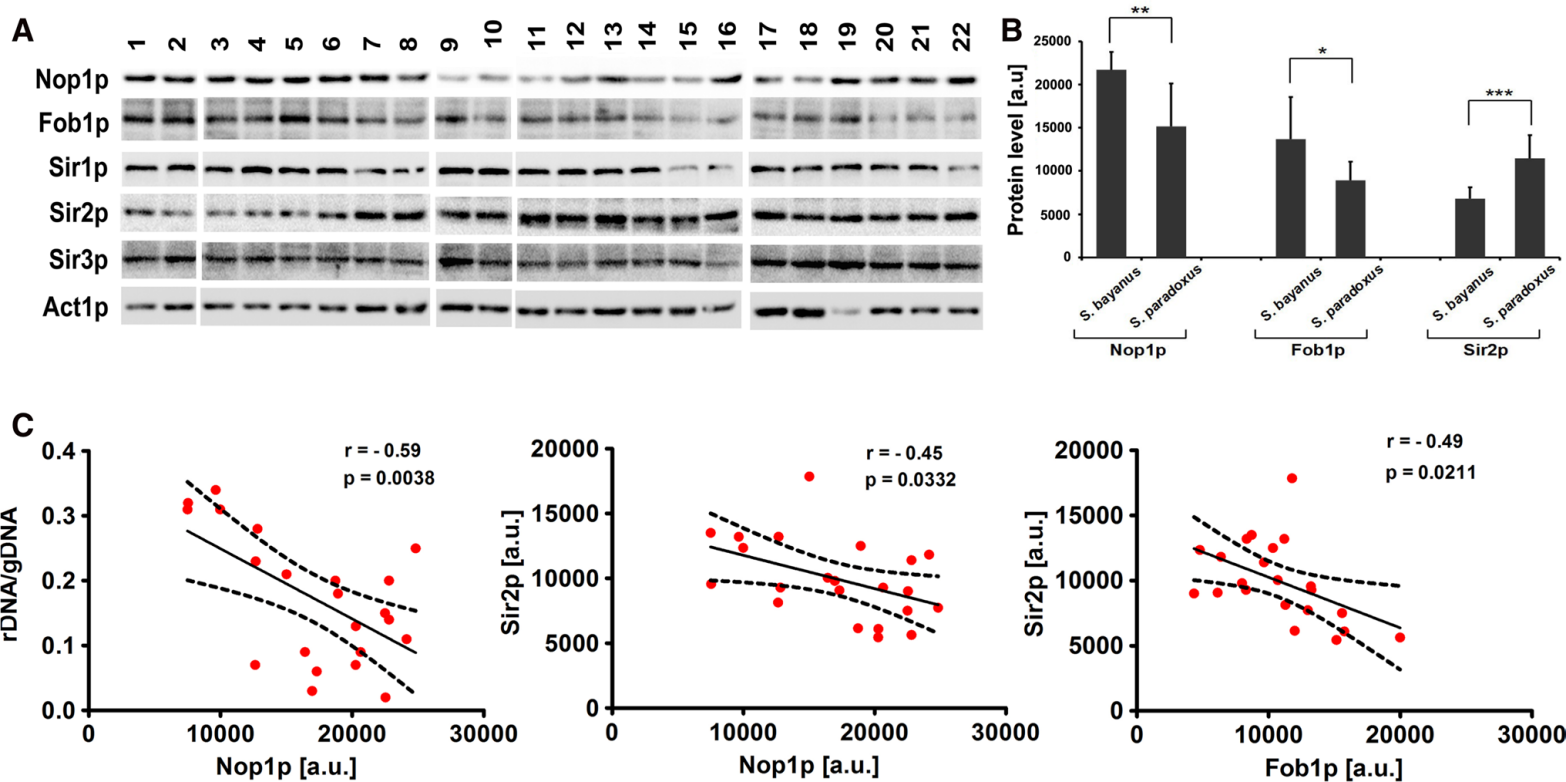
**a** Western blot analysis of Nop1, Fob1, Sir1, Sir2 and Sir3 protein contents in 22 distillery yeasts (*lanes* from 1 to 22). Anti-Act1 antibody served as a loading control. **b** Comparative analysis of Nop1, Fob1 and Sir2 protein levels between two distillery yeast groups, namely *S. bayanus* group (strains 1, 2, 3, 4, 5, 6 and 16) and *S. paradoxus* group (strains 7, 8, 10, 11, 12, 13, 14, 15, 17, 18, 20, 21 and 22). The *bars* indicate SD, *n* = 3, *p < 0.05, **p < 0.01, ***p < 0.001 compared to *S. bayanus* group (Student’s *t* test). **c** Correlation analysis between the length of rDNA and Nop1 (*r* = −0.59, *p* = 0.0038), Sir2 and Nop1 (*r* = −0.45, *p* = 0.0332) and Sir2 and Fob1 (*r* = −0.49, *p* = 0.0211). The 95 % confidence interval is shown. Results represent the mean from three independent experiments. Correlation analysis of the data was performed using a linear correlation (Pearson *r*) test. Protein content is presented in arbitrary units (a.u.)

We found that the length and levels of rDNA varied greatly in analyzed strains (Fig. 1a–c). We have considered then the most abundant groups of distillery yeasts, namely *S. bayanus* group (*n* = 7, strains 1, 2, 3, 4, 5, 6 and 16) and *S. paradoxus* group (*n* = 13, strains 7, 8, 10, 11, 12, 13, 14, 15, 17, 18, 20, 21 and 22), and observed higher rDNA levels in *S. paradoxus* group compared to *S. bayanus* group (Fig. 1d). Except for Southern blotting results, the differences of approximately 20–30 % were statistically significant (*p* < 0.01 and *p* < 0.001, Fig. 1d). Moreover, a great heterogeneity of chromosome XII-specific signals was shown that may suggest increased nucleolus fragmentation in strains with elevated chromosome XII-specific signals, especially in *S. paradoxus* group (Fig. 1e) because chromosome XII contains rDNA locus in yeast (Kim et al. 2006; Petes 1979). Indeed, silver staining of nucleolar organizer region-based analysis of nucleolus fragmentation revealed the presence of affected nucleoli that was more evidently observed in the strains from *S. paradoxus* group (Fig. 1f). As it has been repeatedly reported that nucleolus fragmentation is associated with the upregulation of nucleolar proteins, namely Nop1 and/or Nop2 in yeasts (de Beus et al. 1994; Deregowska et al. 2015a; Lewinska et al. 2014a), we analyzed then the level of Nop1 in distillery strains (Fig. 2).

We did not notice a correlation between nucleolus fragmentation and increased level of Nop1 (data not shown). However, diminished pools of rDNA were observed in yeast cells with elevated levels of Nop1 (*r* = −0.59, *p* = 0.0038, Fig.2c).

Increased levels of chromosome XII-specific signals may also suggest increased levels of aneuploidy events in distillery yeasts, especially in *S. paradoxus* group (Fig. 1e). However, we did not observe increased frequency of other chromosome-specific signals, namely chromosome I-, III- and XI-specific signals (Fig. 1e). This is in agreement with the finding that analyzed distillery strains are mainly diploid with limited incidence of aneuploidy (Deregowska et al. 2015b). Of course, industrial strains are considered more genomically and genetically unstable than laboratory strains and their genomes are dynamic and genomic instability may be provoked as an environmental stress response (Ambrona et al. 2005; James et al. 2008), but one should remember that we have performed FISH analysis under the control conditions without providing stress stimuli. We have reported that the genetic stability of analyzed distillery yeasts is promoted by the *YRF1* gene copy number and strains with lower *YRF1* gene (helicases encoded by the Y′ element of subtelomeric regions) dosage are more susceptible to DNA damage, especially in *S. paradoxus* group (Deregowska et al. 2015b). rDNA is considered to be the most unstable region in the yeast genome being highly repetitive and prone to losing copies by homologous recombination among the repeats (Kobayashi 2008). There are two key modulators of rDNA stability in yeasts by the regulation of rDNA copy number, namely a replication fork-blocking protein, Fob1, and Sir2, a histone deacetylase (Kobayashi 2008; Kobayashi et al. 2004), that levels were investigated in the present study (Fig. 2). We found that the protein levels of Fob1 and Sir2 were negatively correlated in distillery yeasts (*r* = −0.49, *p* = 0.0211, Fig. 2c) that may reflect their opposite role in the rDNA copy number maintenance system. Fob1 binds and blocks the replication fork in the replication fork barrier (RFB) in the rDNA that results in DNA double-strand breaks and unequal sister chromatid recombination that is essential for amplification by producing rDNA copy number variations (Kobayashi et al. 2004; Weitao et al. 2003). On the other hand, Sir2 modulates chromatin structure (Fritze et al. 1997) and silences a noncoding bidirectional promoter in the rDNA, E-pro that prevents from the dissociation of the cohesion complex from the rDNA and changes in rDNA copy number (Kobayashi 2008). We also found that the *S. bayanus* group had a higher level of Fob1 than the *S. paradoxus* group of distillery yeasts (*p* < 0.05; Fig. 2b) and the *S. paradoxus* group had a higher level of Sir2 than the *S. bayanus* group (*p* < 0.001; Fig. 2b). In contrast, the levels of Sir1 and Sir3 were more comparable among analyzed strains (Fig. 2a). The yeast silent information regulator (Sir) protein complex is involved in transcriptional silencing and suppression of recombination at telomeres, silent mating-type *loci* and rDNA modulating the repair of DNA double-strand breaks, mitotic cell cycle, meiosis and longevity (Guarente 1999). Among five sirtuin proteins in yeast, Sir2p is thought to be a limiting component in promoting yeast longevity, because increasing the *SIR2* gene dosage extended replicative lifespan in laboratory yeast cells (Kaeberlein et al. 1999). Overexpression of *SIR2* gene also prolonged chronological lifespan and reduced acetate production during winemaking that indicated that Sir2p is a noteworthy factor for the improvement in alcoholic fermentation (Orozco et al. 2013). More recently, Sir3p and Rap1p, DNA-binding transcription regulator that interacts with Sir3p, were found to be relocated from the telomeres to the nucleolus and their protein expression patterns changed during chronological aging in yeast (Lewinska et al. 2014a). The upregulation of Sir3p and downregulation of Rap1p (Lewinska et al. 2014a) may affect the formation of the Sir transcriptional silencing complex leading to protein redistribution from the telomeres to the nucleolus being a part of an adaptive response during yeast aging (Kennedy et al. 1997). We have also analyzed the relationship between Sir2p and Nop1p and found a negative correlation between the levels of Sir2p and Nop1p in distillery yeasts (*r* = −0.45, *p* = 0.0332, Fig. 2c). It is worthwhile to note that Nop1p may also be considered as an epigenetic regulator in yeast (Tessarz et al. 2014). Nop1p, an ortholog of mammalian fibrillarin involved in pre-rRNA processing (Tollervey et al. 1991), is a histone glutamine methyltransferase that modifies H2A at Q105 and modulates the FACT (facilitator of chromatin transcription) interaction with nucleosomes (Tessarz et al. 2014).

**Fig. 3:**
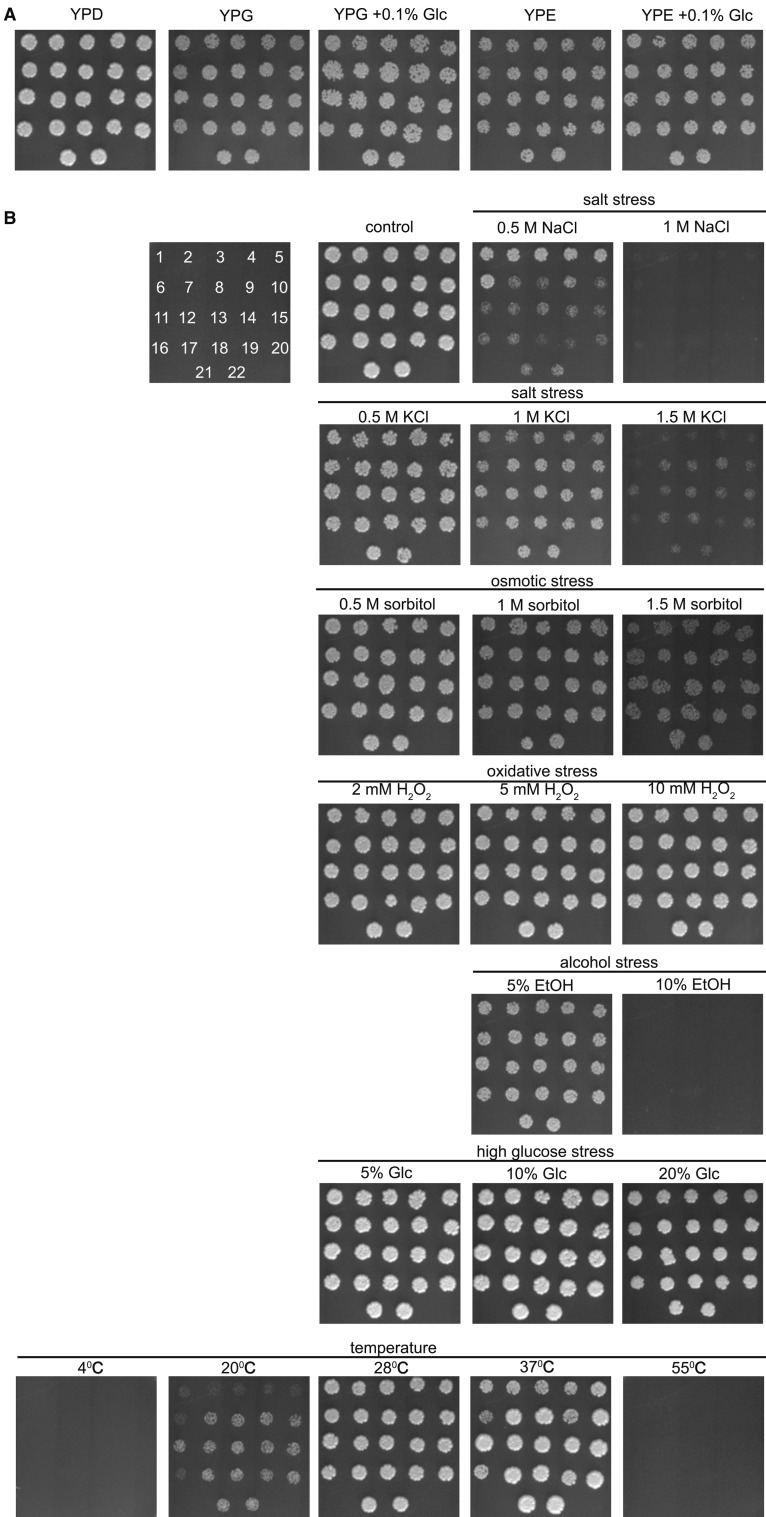
Analysis of the utilization of non fermentable carbon sources (**a**) and sensitivity to fermentation-associated stress stimuli (**b**) in distillery yeasts (strains from 1–22 as denoted on a scheme) using spot assay. **a** Yeast cells at the logarithmic phase of growth were diluted (1 × 10^5^ cells/ml), and growth on solid YPG and YPE media was inspected after 48 h. **b** Yeast cells at the logarithmic phase of growth were diluted (1 × 10^5^ cells/ml), and growth on solid YPD medium in the presence of different stress stimuli was inspected after 48 h. In the case of hydrogen peroxide, cells were incubated with hydrogen peroxide for 40 min and then transferred to solid YPD medium. Representative photographs are shown. Strains 1, 2, 3, 4, 5, 6 and 16, *S. bayanus*; strains 7, 8, 10, 11, 12, 13, 14, 15, 17, 18, 20, 21 and 22, *S. paradoxus*; strain 9, *S. cerevisiae*; strain 19, *S. kudriavzevii*

As we have revealed that two the most abundant groups of distillery yeasts, namely *S. bayanus* group (*n* = 7, strains 1, 2, 3, 4, 5, 6 and 16) and *S. paradoxus* group (*n* = 13, strains 7, 8, 10, 11, 12, 13, 14, 15, 17, 18, 20, 21 and 22), varied in rDNA pools and Sir2 levels, we decided then to analyze the relationship between rDNA content and the sensitivity to fermentation-associated stress stimuli. We have performed a spot assay as this simple platting method has been already considered as an approximation to the true stress conditions during fermentation (Belloch et al. 2008). When fermentation begins, industrial yeast cells are subjected to osmotic stress due to high sugar concentration and low pH (below 4), and as fermentation progresses other stress conditions as ethanol accumulation and nutrient limitation become relevant (Belloch et al. 2008; Cardona et al. 2007). Depending on specific fermentation procedures, other stressors such as high or low temperature may also occur (Belloch et al. 2008; Cardona et al. 2007). First, we have analyzed the utilization of non fermentable carbon sources, namely glycerol and ethanol (Fig. 3a).

The growth capacity of distillery strains was comparable in YPG and YPE media containing glycerol and ethanol as a sole carbon source, respectively (Fig. 3a). The growth was not improved when YPG or YPE media were supplemented with 0.1 % glucose (Fig. 3a). Perhaps, reactive oxygen species (ROS) generated during mitochondrial respiratory chain-based oxidative phosphorylation did not hamper significantly the growth of distillery strains in the presence of glycerol and/or ethanol as a sole carbon source (Fig. 3a). Of course, the maximum growth was observed when distillery strains were cultured in the control conditions, namely in the presence of 2 % glucose (YPD medium) (Fig. 3a). Second, the tolerance to fermentation-associated stress stimuli was considered, namely salt, osmotic, oxidative, ethanol, high glucose and cold/heat stresses (Fig. 3b). In general, the resistance to KCl treatment (hyperosmotic stress) and mild cold/heat stresses was increased in *S. paradoxus* group compared to *S. bayanus* group (Fig. 3b). Perhaps, this tolerance may be correlated with higher levels of rDNA and Sir2p in *S. paradoxus* group (Figs. 1d and 2b). On the other hand, *S. bayanus* group was found to be more tolerant to NaCl treatment compared to *S. paradoxus* group that may reflect ion-dependent response to hyperosmotic stress. More recently, we have also revealed that rDNA (Deregowska et al. 2015a) and sirtuins (Adamczyk et al. 2016) are the regulators of adaptive response to chronic mild ethanol stress in wine yeast strains.

In summary, we have characterized for the first time the nucleolus state, namely the rDNA level and length, the nucleolus fragmentation, the level of nucleolar protein Nop1 and the regulators of rDNA stability, Fob1p and Sir2p in distillery yeasts. As we have recently reported that rDNA may regulate genomic stability and control cellular stress response in industrially relevant yeast strains (Deregowska et al. 2015a) and rDNA content may reflect the tolerance to fermentation-associated stress stimuli (this study), it seems worthwhile to consider rDNA state as a novel factor that may modulate yeast cell vitality and viability, and fermentation efficiency during biotechnological processes at harsh environmental conditions.

## References

[CR1] Adamczyk J (2016). Adaptive response to chronic mild ethanol stress involves ROS, sirtuins and changes in chromosome dosage in wine yeasts. Oncotarget.

[CR2] Amberg DC, Burke DJ, Strathern JN (2005). Methods in yeast genetics: a cold spring harbor laboratory course manual.

[CR3] Ambrona J, Vinagre A, Ramirez M (2005). Rapid asymmetrical evolution of *Saccharomyces cerevisiae* wine yeasts. Yeast.

[CR4] Belloch C, Orlic S, Barrio E, Querol A (2008). Fermentative stress adaptation of hybrids within the *Saccharomyces sensu stricto* complex. Int J Food Microbiol.

[CR5] Boisvert FM, van Koningsbruggen S, Navascues J, Lamond AI (2007). The multifunctional nucleolus. Nat Rev Mol Cell Biol.

[CR6] Cardona F, Carrasco P, Perez-Ortin JE, del Olmo M, Aranda A (2007). A novel approach for the improvement of stress resistance in wine yeasts. Int J Food Microbiol.

[CR7] Covington JD, Bajpeyi S (2016). The sirtuins: markers of metabolic health. Mol Nutr Food Res.

[CR8] de Beus E, Brockenbrough JS, Hong B, Aris JP (1994). Yeast *NOP2* encodes an essential nucleolar protein with homology to a human proliferation marker. J Cell Biol.

[CR9] Defossez PA (1999). Elimination of replication block protein Fob1 extends the life span of yeast mother cells. Mol Cell.

[CR10] Deregowska A (2015). Shifts in rDNA levels act as a genome buffer promoting chromosome homeostasis. Cell Cycle.

[CR11] Deregowska A (2015). Genome-wide array-CGH analysis reveals *YRF1* gene copy number variation that modulates genetic stability in distillery yeasts. Oncotarget.

[CR12] Fritze CE, Verschueren K, Strich R, Esposito RE (1997). Direct evidence for *SIR2* modulation of chromatin structure in yeast rDNA. EMBO J.

[CR13] Giblin W, Skinner ME, Lombard DB (2014). Sirtuins: guardians of mammalian healthspan. Trends Genet.

[CR14] Grummt I (2013). The nucleolus—guardian of cellular homeostasis and genome integrity. Chromosoma.

[CR15] Guarente L (1999). Diverse and dynamic functions of the Sir silencing complex. Nat Genet.

[CR16] James TC, Usher J, Campbell S, Bond U (2008). Lager yeasts possess dynamic genomes that undergo rearrangements and gene amplification in response to stress. Curr Genet.

[CR17] Kaeberlein M, McVey M, Guarente L (1999). The *SIR2/3/4* complex and *SIR2* alone promote longevity in *Saccharomyces cerevisiae* by two different mechanisms. Genes Dev.

[CR18] Kennedy BK (1997). Redistribution of silencing proteins from telomeres to the nucleolus is associated with extension of life span in *S. cerevisiae*. Cell.

[CR19] Kim YH, Ishikawa D, Ha HP, Sugiyama M, Kaneko Y, Harashima S (2006). Chromosome XII context is important for rDNA function in yeast. Nucleic Acids Res.

[CR20] Kobayashi T (2006). Strategies to maintain the stability of the ribosomal RNA gene repeats—collaboration of recombination, cohesion, and condensation. Genes Genet Syst.

[CR21] Kobayashi T (2008). A new role of the rDNA and nucleolus in the nucleus—rDNA instability maintains genome integrity. BioEssays.

[CR22] Kobayashi T, Horiuchi T, Tongaonkar P, Vu L, Nomura M (2004). *SIR2* regulates recombination between different rDNA repeats, but not recombination within individual rRNA genes in yeast. Cell.

[CR23] Lewinska A, Wnuk M, Grzelak A, Bartosz G (2010). Nucleolus as an oxidative stress sensor in the yeast *Saccharomyces cerevisiae*. Redox Rep.

[CR24] Lewinska A, Macierzynska E, Grzelak A, Bartosz G (2011). A genetic analysis of nitric oxide-mediated signaling during chronological aging in the yeast. Biogerontology.

[CR25] Lewinska A, Miedziak B, Kulak K, Molon M, Wnuk M (2014). Links between nucleolar activity, rDNA stability, aneuploidy and chronological aging in the yeast *Saccharomyces cerevisiae*. Biogerontology.

[CR26] Lewinska A, Miedziak B, Wnuk M (2014). Assessment of yeast chromosome XII instability: single chromosome comet assay. Fungal Genet Biol.

[CR27] Mayer C, Grummt I (2005). Cellular stress and nucleolar function. Cell Cycle.

[CR28] Mayer C, Zhao J, Yuan X, Grummt I (2004). mTOR-dependent activation of the transcription factor TIF-IA links rRNA synthesis to nutrient availability. Genes Dev.

[CR29] Mayer C, Bierhoff H, Grummt I (2005). The nucleolus as a stress sensor: JNK2 inactivates the transcription factor TIF-IA and down-regulates rRNA synthesis. Genes Dev.

[CR30] Naumova ES, Sadykova AZ, Martynenko NN, Naumov GI (2013). Molecular genetic characteristics of *Saccharomyces cerevisiae* distillers’ yeasts. Microbiology.

[CR31] Olson MO (2004). Sensing cellular stress: another new function for the nucleolus?. Sci STKE.

[CR32] Orozco H, Matallana E, Aranda A (2013). Genetic manipulation of longevity-related genes as a tool to regulate yeast life span and metabolite production during winemaking. Microb Cell Factories.

[CR33] Pederson T (1998). The plurifunctional nucleolus. Nucleic Acids Res.

[CR34] Petes TD (1979). Yeast ribosomal DNA genes are located on chromosome XII. Proc Natl Acad Sci USA.

[CR35] Rubbi CP, Milner J (2003). Disruption of the nucleolus mediates stabilization of p53 in response to DNA damage and other stresses. EMBO J.

[CR36] Tessarz P, Santos-Rosa H, Robson SC, Sylvestersen KB, Nelson CJ, Nielsen ML, Kouzarides T (2014). Glutamine methylation in histone H2A is an RNA-polymerase-I-dedicated modification. Nature.

[CR37] Tollervey D, Lehtonen H, Carmo-Fonseca M, Hurt EC (1991). The small nucleolar RNP protein NOP1 (fibrillarin) is required for pre-rRNA processing in yeast. EMBO J.

[CR38] Wai HH, Vu L, Oakes M, Nomura M (2000). Complete deletion of yeast chromosomal rDNA repeats and integration of a new rDNA repeat: use of rDNA deletion strains for functional analysis of rDNA promoter elements in vivo. Nucleic Acids Res.

[CR39] Weitao T, Budd M, Hoopes LL, Campbell JL (2003). Dna2 helicase/nuclease causes replicative fork stalling and double-strand breaks in the ribosomal DNA of *Saccharomyces cerevisiae*. J Biol Chem.

[CR40] Wnuk M, Lewinska A, Bugno M, Bartosz G, Slota E (2009). Rapid detection of yeast rRNA genes with primed in situ (PRINS) labeling. FEMS Yeast Res.

[CR41] Wnuk M (2015). Single-cell analysis of aneuploidy events using yeast whole chromosome painting probes (WCPPs). J Microbiol Methods.

